# Young Woman Presents With Abdominal Pain

**DOI:** 10.1016/j.acepjo.2026.100445

**Published:** 2026-06-12

**Authors:** Raymond Che, Allyson M. Briggs, Alex Hodson, Avery Miller

**Affiliations:** Department of Emergency Medicine, University of Kansas Health System and University of Kansas Medical Center, Kansas City, Kansas, USA

**Keywords:** small bowel obstruction, mechanical obstruction, jejunostomy tube balloon

## 1 Introduction

**Figure 1:**
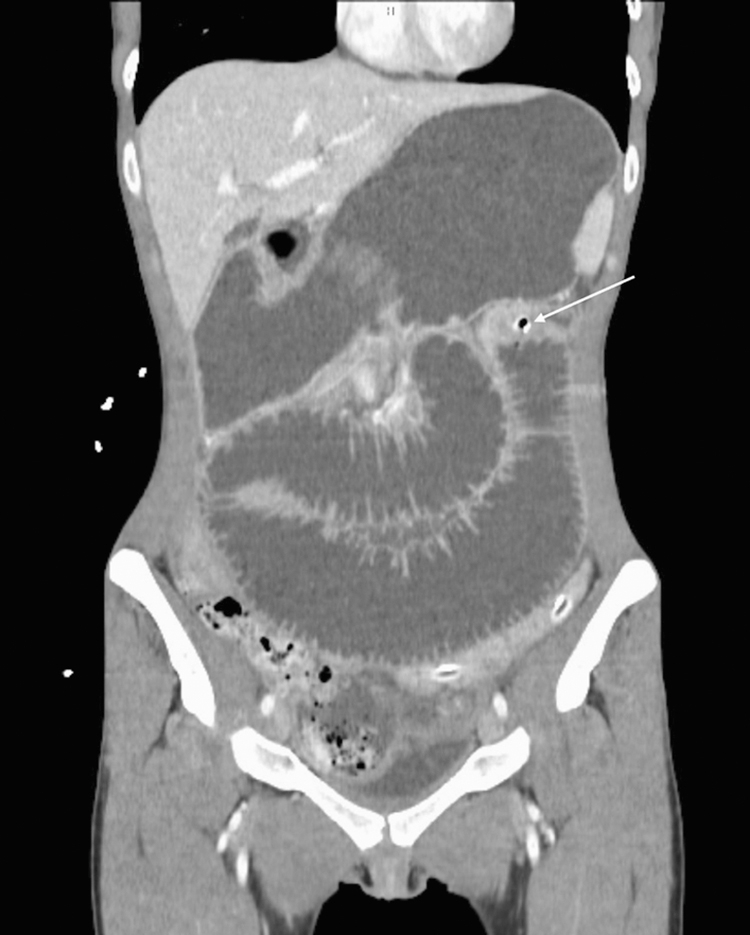
Coronal computed tomography demonstrating gastric and proximal small bowel dilation with a transition point associated with a jejunostomy tube balloon (arrow); jejunal loops measure up to 6 cm.

**Figure 2:**
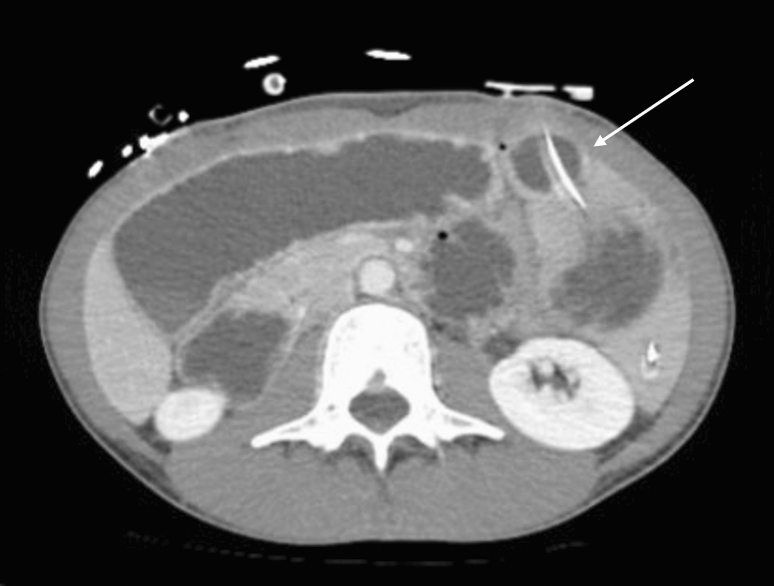
Axial computed tomography demonstrating dilation of the proximal jejunum and duodenum adjacent to the jejunostomy tube balloon (arrow).

A 28-year-old woman presented with abdominal pain, nausea, vomiting, and distension. Her history was significant for anorexia nervosa and gastroparesis, with recent jejunostomy tube placement for nutritional support. She was tachycardic, but otherwise hemodynamically stable. Examination revealed a distended abdomen with diffuse tenderness, and the jejunostomy tube site was intact. Laboratory evaluation was unremarkable. Computed tomography of the abdomen and pelvis demonstrated marked gastric and proximal small bowel dilation with a transition point at the jejunostomy tube balloon (Figs. 1 and 2).

**Figure 3:**
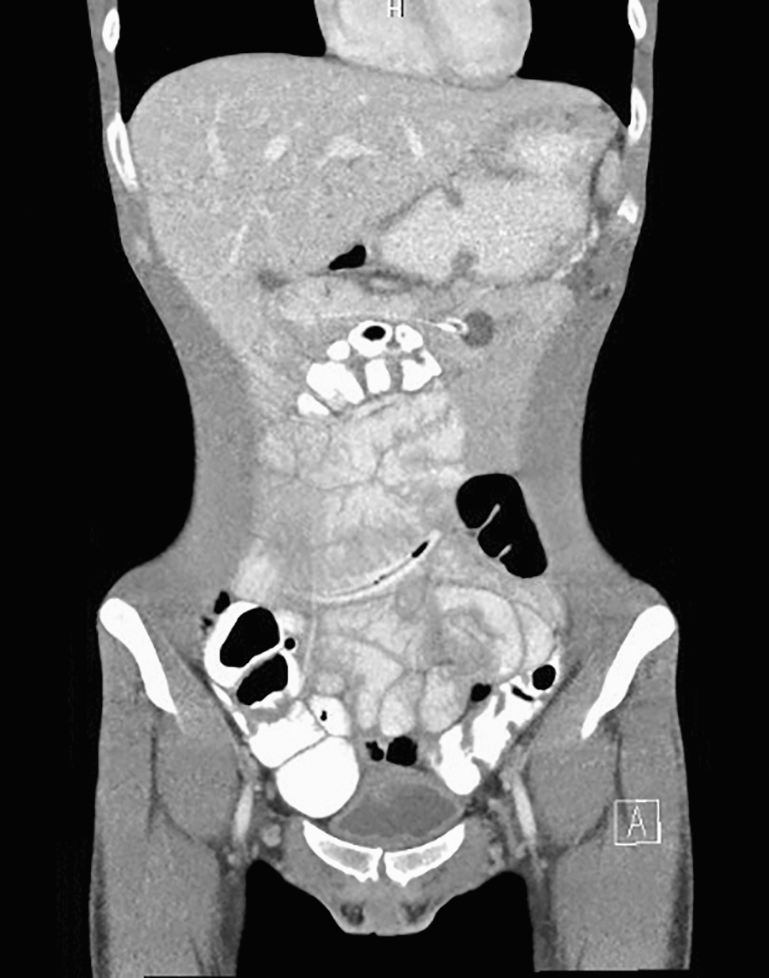
Coronal computed tomography demonstrating resolution of small bowel obstruction following balloon deflation and gastric decompression.

**Figure 4:**
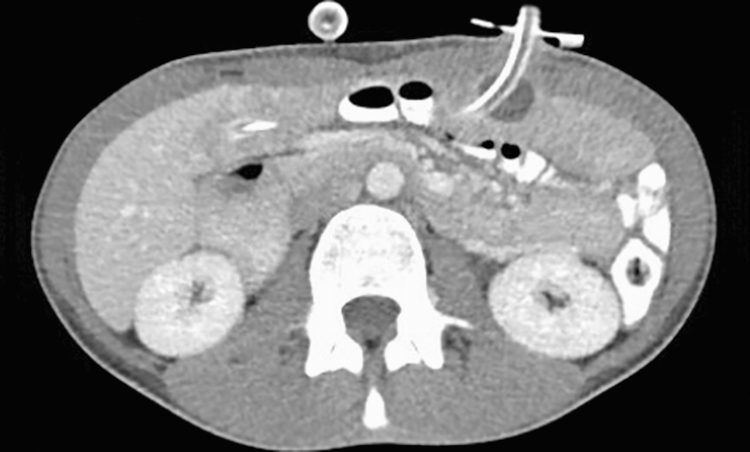
Axial computed tomography demonstrating resolution of small bowel obstruction following balloon deflation and gastric decompression.

Initial management included deflation of the balloon by 1.5 mL from the reported 7 mL, resulting in rapid symptom resolution. The patient was discharged but returned 4 days later with recurrent obstruction. Repeat evaluation confirmed recurrent obstruction, and an additional 2.5 mL of fluid was removed from the balloon with complete resolution (Figs. 3 and 4).

## 2 Diagnosis: Mechanical Small Bowel Obstruction From Jejunostomy Tube Balloon

Mechanical obstruction from feeding tube balloons is a rare complication described in prior case reports.^1^, ^2^, ^3^ These devices may cause obstruction through balloon overdistension or migration, resulting in luminal narrowing and impaired passage of intestinal contents. In this case, partial balloon deflation resulted in transient improvement, with recurrence requiring additional volume reduction for definitive resolution. Recognition in the emergency department is important, as this represents a reversible etiology that may be managed nonoperatively.^4^

## Funding and Support

By *JACEP Open* policy, all authors are required to disclose any and all commercial, financial, and other relationships in any way related to the subject of this article as per ICMJE conflict of interest guidelines (see www.icmje.org). The authors have stated that no such relationships exist.

## Conflict of Interest

All authors have affirmed they have no conflicts of interest to declare.
